# Corrigendum to “Sequence Analysis of the *UCP1* Gene in a Severe Obese Population from Southern Italy”

**DOI:** 10.1155/2018/3260210

**Published:** 2018-07-19

**Authors:** Giuseppe Labruna, Fabrizio Pasanisi, Giuliana Fortunato, Carmela Nardelli, Carmine Finelli, Eduardo Farinaro, Franco Contaldo, Lucia Sacchetti

**Affiliations:** ^1^Fondazione IRCCS SDN, Istituto di Ricerca Diagnostica e Nucleare, Via Gianturco 113, 80143 Naples, Italy; ^2^Centro Interuniversitario di Studi e Ricerche sull'Obesità e Dipartimento di Medicina Clinica e Sperimentale, Università degli Studi di Napoli Federico II, Via Pansini 5, 80131 Naples, Italy; ^3^CEINGE Biotecnologie Avanzate S.C. a R.L., Via Gaetano Salvatore 486, 80145 Naples, Italy; ^4^Dipartimento di Biochimica e Biotecnologie Mediche, Università degli Studi di Napoli Federico II, Via Pansini 5, 80131 Naples, Italy; ^5^Independent Researcher, Naples, Italy; ^6^Dipartimento di Scienze Mediche Preventive, Università degli Studi di Napoli Federico II, Via Pansini 5, 80131 Naples, Italy

In the article titled “Sequence Analysis of the *UCP1* Gene in a Severe Obese Population from Southern Italy” [1], the fifth author, Carmine Finelli, is not affiliated to the Fondazione Stella Maris Mediterraneo and should be listed as an independent researcher. The corrected list of affiliations is shown above.
